# Functional and structural alterations in different durations of untreated illness in the frontal and parietal lobe in major depressive disorder

**DOI:** 10.1007/s00406-023-01625-7

**Published:** 2023-08-05

**Authors:** Wen Liu, Xiaowei Jiang, Zijing Deng, Yu Xie, Yingrui Guo, Yifan Wu, Qikun Sun, Lingtao Kong, Feng Wu, Yanqing Tang

**Affiliations:** 1 Brain Function Research Section, The First Hospital of China Medical University, Shenyang, 110001 Liaoning People’s Republic of China; 2 Department of Psychiatry, The First Hospital of China Medical University, Shenyang, 110001 Liaoning People’s Republic of China; 3 Department of Radiology, The First Hospital of China Medical University, Shenyang, 110001 Liaoning People’s Republic of China; 4 Department of Radiation Oncology, The First Hospital of China Medical University, Shenyang, 110001 Liaoning People’s Republic of China; 5 Department of Gerontology, The First Hospital of China Medical University, Shenyang, 110001 Liaoning People’s Republic of China; 6 Department of Psychiatry and Geriatric Medicine, The First Hospital of China Medical University, 155 Nanjing North Street, Shenyang, 110001 Liaoning People’s Republic of China

**Keywords:** Major depressive disorder, Duration of untreated illness, Regional homogeneity, Voxel-based morphometry, Grey matter volume

## Abstract

Major depressive disorder (MDD) is one of the most disabling illnesses that profoundly restricts psychosocial functions and impairs quality of life. However, the treatment rate of MDD is surprisingly low because the availability and acceptability of appropriate treatments are limited. Therefore, identifying whether and how treatment delay affects the brain and the initial time point of the alterations is imperative, but these changes have not been thoroughly explored. We investigated the functional and structural alterations of MDD for different durations of untreated illness (DUI) using regional homogeneity (ReHo) and voxel-based morphometry (VBM) with a sample of 125 treatment-naïve MDD patients and 100 healthy controls (HCs). The MDD patients were subgrouped based on the DUI, namely, DUI ≤ 1 M, 1 < DUI ≤ 6 M, 6 < DUI ≤ 12 M, and 12 < DUI ≤ 48 M. Subgroup comparison (MDD with different DUIs) was applied to compare ReHo and grey matter volume (GMV) extracted from clusters of regions with significant differences (the pooled MDD patients relative to HCs). Correlations and mediation effects were analysed to estimate the relationships between the functional and structural neuroimaging changes and clinical characteristics. MDD patients exhibited decreased ReHo in the left postcentral gyrus and precentral gyrus and reduced GMV in the left middle frontal gyrus and superior frontal gyrus relative to HCs. The initial functional abnormalities were detected after being untreated for 1 month, whereas this duration was 3 months for GMV reduction. Nevertheless, a transient increase in ReHo was observed after being untreated for 3 months. No significant differences were discovered between HCs and MDD patients with a DUI less than 1 month or among MDD patients with different DUIs in either ReHo or GMV. Longer DUI was related to reduced ReHo with GMV as mediator in MDD patients. We identified disassociated functional and anatomical alterations in treatment-naïve MDD patients at different time points in distinct brain regions at the early stage of the disease. Additionally, we also discovered that GMV mediated the relationship between a longer DUI and diminished ReHo in MDD patients, disclosing the latent deleterious and neuro-progressive implications of DUI on both the structure and function of the brain and indicating the necessity of early treatment of MDD.

**Supplementary Information:**

The online version contains supplementary material available at 10.1007/s00406-023-01625-7.

## Introduction

Major depressive disorder (MDD) is one of the most disabling illnesses [1] that profoundly restricts psychosocial functions and impairs quality of life [2], with an overall 12-month prevalence of 6% [3]. However, the treatment rate of MDD is surprisingly low, ranging from 20 to 51% across 84 countries [4], and a meta-analysis covering 609,054 participants indicated that the pooled treatment rate for MDD in China was 19.5% [5]. In addition to the low availability of the treatments, poor acceptability is also an obstacle to appropriate treatments [6–8]. As a result, identifying whether and how treatment delay may affect MDD patients is imperative, but these changes have not been thoroughly explored.

The duration of untreated illness (DUI) refers to the interval between the first onset of an episode and the administration of appropriate treatment [9]. Altamura et al. contended that a longer DUI was related to a worse clinical course, including a longer duration of illness, earlier age of onset, higher recurrence rate, and greater comorbidity rate with other mental disorders [9], even after 5 years of follow-up [10]. Previous studies observed significantly greater odds of response and remission, as well as steeper declines in disability ratings, in patients with both first-episode and recurrent depression who exhibited a shorter DUI [11–13]. A longer DUI has also been reported to be associated with worse clinical outcomes [14] in various psychiatric diseases, including worse cognitive performance during depression [15], greater severity and lower response rate to medication [16, 17], more episodes and suicidal behaviours and longer lifetime mood fluctuations [18], but no consistent conclusions have been drawn [19, 20].

Functional and structural abnormalities have been frequently reported in first-episode, drug-naïve MDD [21–25], indicating an early-stage alteration of the brain. However, studies of the implications of DUI on neuroimaging alterations in MDD are limited, especially in the resting state. Smaller hippocampal grey matter volumes (GMVs) were observed in MDD, which was inversely correlated with DUI [26]. MacQueen et al. also proposed a mediation effect of greater episodes of untreated illness on smaller subcallosal gyrus volumes in MDD patients compared with healthy controls (HCs) [27], implying a latent deleterious impact of DUI on brain structures in MDD. However, a small-sample study identified GMV reduction in the bilateral limbic system in first-episode, drug-naïve MDD but observed no correlations between DUI and reduced GMV [28]. Studies of psychosis also failed to draw a congruous conclusion on the relationships between DUI and brain alterations. For example, a longer DUI was found to be related to lower cortical activities [29], reduced functional connectivity [30], and decreased GMV in schizophrenia [31] and first-episode psychosis, including schizophrenia and schizophreniform disorder [32]. Nevertheless, anatomical and functional abnormalities were reported to be independent of DUI in other studies [33, 34]. Thus, more studies with a larger sample size are required to investigate the functional and structural implications of DUI on the brain in MDD.

Resting-state fMRI (rs-fMRI) is a promising approach to describe the regional functional activities of the brain, in which regional homogeneity (ReHo) is a model-driven method that is sensitive to unpredicted haemodynamic responses [35]. Voxel-based morphometry (VBM) is a technique to detect GMV modifications on a voxel-by-voxel basis [36]. The two approaches have been widely applied to explore anatomical and functional abnormalities in various psychiatric disorders. Whether and how DUI affects the brain functionally and anatomically in MDD remains to be elucidated due to the limited research and discrepant conclusions of previous studies. Therefore, we aimed to characterize the alterations in ReHo and GMV in MDD patients with different DUIs and identify the initial time point at which they occurred.

## Methods

### Subjects

We recruited 141 treatment-naïve (never took medication or accepted nonpharmacological interventions) MDD patients with no psychotic symptoms or other mental disorders from the Department of Psychiatry at the First Hospital of China Medical University and Shenyang Mental Health Centre. All patients were diagnosed by experienced psychiatrists and were in a depressive episode at the time of the scan. For the validity of the diagnosis, well-trained researchers further interviewed participants ≥ 18 years of age with the Structured Clinical Interview for DSM-IV (SCID) [37] and participants < 18 years of age with the Schedule for Affective Disorders and Schizophrenia for School-Age Children. 16 patients were excluded for quality control after preprocessing, out of head motion, artifact, or lacking parietal or temporal lobe, and 125 patients were included for final neuroimaging analysis. To investigate whether the brain alters in function and structure according to the DUI, and to equally distribute MDD participants in different subgroups as possible as we could, all patients were then allocated to 4 groups: DUI ≤ 1 M (*n* = 25), 1 < DUI ≤ 6 M (*n* = 44), 6 < DUI ≤ 12 M (*n* = 29), 12 < DUI ≤ 48 M (*n* = 27). To identify the initial time point of the alteration, the subgroup with the earliest time point was then reallocated.

Through advertisements, we also recruited 105 healthy controls with neither a personal nor family history of mental illness who matched to the MDD group in age, gender, handedness, and years of education, and 100 were included for data analysis after quality control. The participants were appraised with the Young Mania Rating Scale (YMRS) [38], 17-item Hamilton Depression Rating Scale (HAMD-17) [39], Hamilton Anxiety Rating Scale (HAMA) [40], and Wisconsin Card Sorting Test (WCST) [41] at the time of scanning. However, only 97 MDD patients and 80 HCs completed the full WCST.

All participants were 15–60 years old, with no neurological diseases and no history of head trauma with loss of consciousness ≥ 3 min. Those with major medical conditions, medications that may affect mental health or those with contradictions for MRI scanning were excluded. Written informed consent was obtained from all participants. For those who were under 18 years, written consent was gained from both the participants and their parents. The study was approved by the Ethics Committee of the First Hospital of China Medical University.

### MRI acquisition

All participants were scanned on the same GE SIGNA HD 3.0 T MRI scanner with a standard eight-channel head coil at the First Hospital of China Medical University, Shenyang, China. Foam pads and earplugs were offered to all the participants to minimize head motion and noise during scanning. All participants were instructed to close their eyes and stay awake during the scan. The rs-fMRI parameters were 200 echo-planar imaging (EPI) volumes; TR = 2000 ms; TE = 40 ms; flip angle = 90°; field of view (FOV) = 24 × 24 cm; acquisition matrix = 64 × 64; slice thickness = 3 mm; spacing between slices = 3 mm; slices = 35, and scan time = 400 s. A three-dimensional fast spoiled gradient-echo sequence was applied to acquire a three-dimensional, high-resolution T1-weighted anatomical image with TR/TE = 7.148/3.15 ms, flip angle = 13^°^, FOV = 240 mm × 240 mm, matrix size = 24 × 24 cm, slice thickness = 1 mm, slice spacing = 1 mm, and voxel size = 1.0 × 1.0 × 1.0 mm^3^.

### fMRI data preprocessing and ReHo calculation

The rs-fMRI functional data were preprocessed using DPABI [42]. The first 10 images were discarded to ensure that the signal reached equilibrium. The remaining 190 images were then corrected for different timings between slices. Realignment was applied for head movement correction. Participants were only included when head motion was < 3 mm and rotation was < 3° in each direction. The Montreal Neurological Institute (MNI) EPI template was applied to the functional images for normalization, after which the images were resampled to 3 × 3 × 3 mm^3^. Nuisance covariates were regressed out from the functional signal, including 24 head motion parameters [43], white matter signal, cerebrospinal fluid signal, and linear trend. To minimize the impact of high-frequency psychological noise and very low-frequency drift, bandpass filtering (0.01–0.08 Hz) was conducted. The ReHo of the time series of a given voxel and the ones around it (26 voxels) were measured with Kendall’s coefficient of concordance (KCC) in a voxelwise manner [35]. A group mask was applied to exclude the nonbrain tissues. Each individual ReHo map was divided by the global mean KCC within the mask for standardization [44]. The standardized ReHo maps were spatially smoothed with a 6-mm FWHM Gaussian kernel.

### Voxel-based morphometry

For structural MRI data, the VBM8 toolbox (http://dbm.neuro.uni-jena.de/vbm8/) was integrated into Statistical Parametric Mapping (SPM 8, Wellcome Department of Cognitive Neurology, London, UK. www.fil.ion.ucl.ac.uk/spm/software/spm8/). According to Ashburner [45], the structural images were spatially normalized to MNI using Diffeomorphic Anatomical Registration through Exponentiated Lie (DARTEL) algebra [46], and affine registration [47] was performed with ICBM space template-East Asian Brains. The normalized images were then segmented into grey matter, white matter, and cerebrospinal fluid, subjected to data denoising [48] and nonlinear modulation, and resampled to images with 1.5 mm^3^ isotropic voxels. Subsequently, the normalized images were smoothed with an 8-mm full width at half-maximum (FWHM) Gaussian kernel. Computational Anatomy Toolbox (CAT 12) was used for total intracranial volume (TIV) calculation.

### Statistical analysis

SPSS v26.0 (SPSS, Chicago, IL, USA) was used for statistical analyses. To compare the demographic and clinical data between MDD patients and HCs, we performed independent *t test*s and *χ*^*2*^ tests for continuous variables and categorical variables, respectively. Analyses of variance (ANOVA) and *t test*s were conducted to compare demographic and clinical variables among the subgroups allocated according to the DUI, with Bonferroni correction for post hoc analysis. The statistical significance threshold was set to *p* < 0.05. DPABI v 6.0 was applied to assess group differences in ReHo and GMV between MDD patients and HCs using general linear models, with covariates of TIV, age, age^2^, gender, and years of education. The statistical significance threshold was set to *p* < 0.001 with Gaussian random field (GRF) theory for correction at the voxel level, and the threshold for the cluster level was set to *p* < 0.05. To identify whether functional and structural abnormalities of the brain change over the DUI and the time point when alterations initially occurred, ANOVA for subgroup comparison and Bonferroni post hoc correction were applied to compare ReHo and GMV extracted from clusters of regions with significant differences, with a significance level threshold of *p* < 0.05.

Log transformation was used for skewness management and leveraged data point moderation [29]. The DUI values were transformed to the base 10 logarithm (log_10_ DUI). The distribution of skewness of DUI (skewness: 1.698, kurtosis: 3.105) was lessened to that of log_10_ DUI (skewness: -0.113, kurtosis: -1.127) after log transformation, after which Pearson and partial correlations were conducted to estimate the relationships between functional and structural modifications and clinical characteristics, and false discovery rate (FDR) was used for correction. Finally, a mediation analysis was applied to investigate the relationship between DUI, GMV and ReHo, in which log_10_ DUI was taken as an independent variable, GMV value as a mediator, and ReHo value as a dependent variable. According to Zhao et al. [49], a nonparametric bootstrap analysis [50] was performed, in which the mediation was significant if 0 was not included in the confidence intervals (CIs) with 95% bias correction.

## Results

### Clinical and demographic characteristics

The mean age of the MDD group and HC group was 26.99 years and 28.51 years respectively, and the mean years of education were 13.72 years for the MDD group and 14.45 years for the HC group. Male participants accounted for 20.80% in the MDD group and 31.00% in the HC group, and most of the participants were right-handed (MDD:94.40%, HC:100%). For MDD participants, the mean DUI was 9.15 months, with 0.67 times as their mean number of previous episodes. The average age of the first onset was 26.18 years old. No significant differences were observed between MDD patients and HCs in age (*p* = 0.226), sex (*p* = 0.080), handedness (*p* = 0.056), or years of education (*p* = 0.056). The total HAMD-17, HAMA, and YMRS scores were significantly higher in MDD patients than in HCs (*p* < 0.001). The mean HAMD-17, HAMA, and YMRS scores in MDD patients were 20.85, 19.18, and 1.22, respectively. For HC participants, the mean HAMD-17, HAMA, and YMRS scores were 1.31, 1.02, and 0.1 respectively. The total WCST scores were significantly lower in MDD patients than in HCs, with a mean score of 29.36 for MDD participants and 36.79 for HC participants (*p* < 0.001, see Table 1). The demographic and clinical features among the MDD subgroups are presented in Supplementary Table 1 and Table 2.

**Table 1:** Demographic and Clinical Characteristics of MDD Patients and HCs

	MDD (*n* = 125)	HC (*n* = 100)	*t*/*χ2*	*p*
*Demographic characteristics*				
Age at scan, years	26.99 (9.38)	28.51 (9.22)	-1.22	0.226
Education, years	13.72 (2.99)	14.45 (2.70)	-1.92	0.056
Male	26 (20.80%)	31 (31.00%)	3.06	0.080
Right handedness	118 (94.40%)	100 (100.00%)	5.78	0.056
* Clinical features*				
Duration of un-treatment, months	9.15 (9.63)	N/A	N/A	N/A
Number of previous episodes, times	0.67 (0.49)	N/A	N/A	N/A
Age of first onset, years	26.18 (9.54)	N/A	N/A	N/A
WCST	29.36 (10.31)	36.79 (6.47)	-5.60	< 0.001
HAMD_17	20.85 (7.78)	1.31 (1.63)	27.34	< 0.001
HAMA	19.18 (8.65)	1.02 (1.65)	22.97	< 0.001
YMRS	1.22 (1.73)	0.1 (0.44)	6.98	< 0.001

Data are presented as either numbers (%) or means (standard deviations). Independent sample *t test* was conducted for continuous variables and chi-square *test* was conducted for categorical variables. Number of participants completed WSCT: MDD: 97; HC: 80

*MDD* Major depressive disorder, *HCs* Healthy controls, *N/A* Not applicable, *WCST* Wisconsin Card Sorting Test, *HAMD-17* 17-Item Hamilton Depression Rating Scale, *HAMA* Hamilton Anxiety Rating Scale, *YMRS* Young Mania Rating Scale

**Table 2:** Clusters Showing Significant Differences in ReHo between MDD Patients and HCs

Brain Regions (voxels)	Contrast	Hemisphere	Peak MNI Coordinates			*t* Values	*p*
			X	Y	Z		
Postcentral gyrus (49)	HC > MDD	Left	-57	-6	30	4.757	< 0.001
Precentral gyrus (21)							

The pooled MDD patients and HCs were compared with 2-tailed *t tests*

ReHo: Regional homogeneity, *MDD* Major depressive disorder, *HCs* Healthy controls, *MNI* Montreal Neurological Institute

### ReHo analysis

#### Pooled MDD versus HCs

**Fig. 1:**
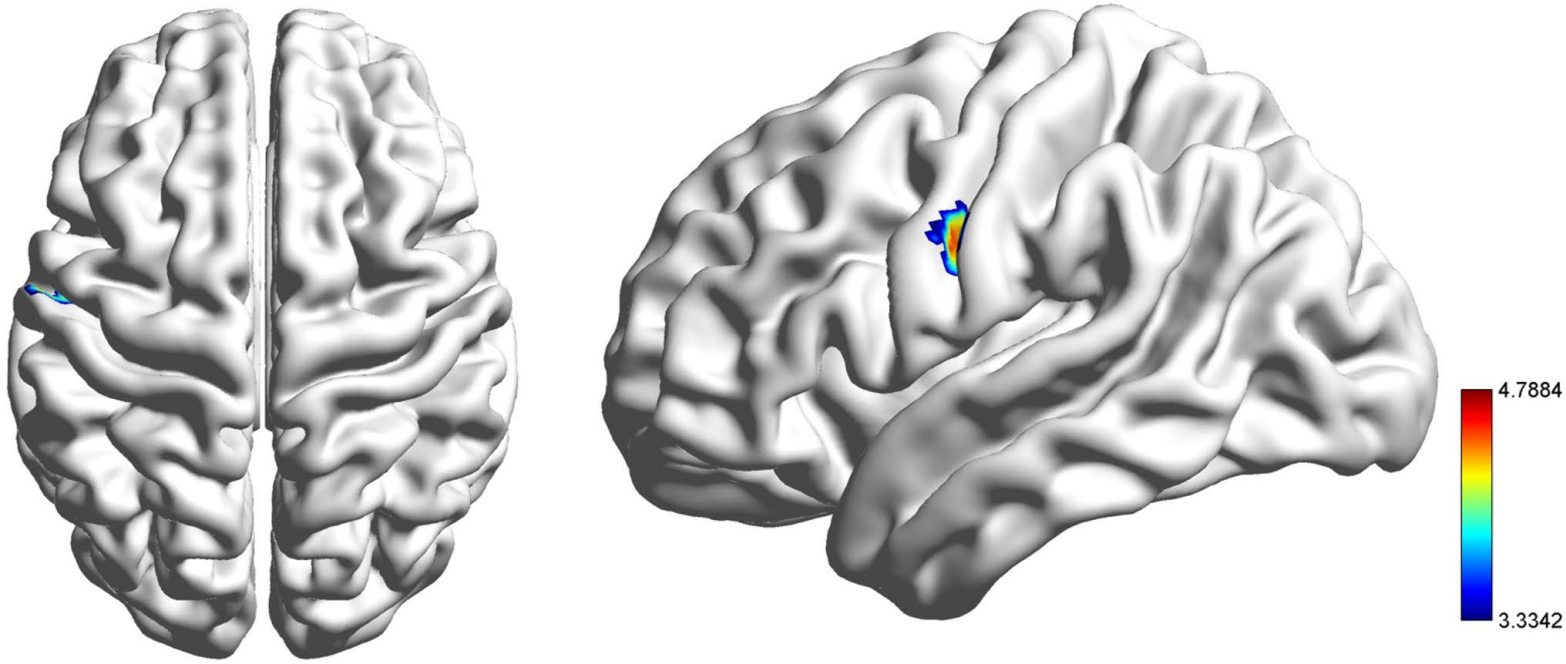
Significantly decreased ReHo in the left postcentral gyrus and precentral gyrus in the pooled MDD patients compared with HCs with 2-tailed* t tests*. The significance of the voxel-level threshold was set to *p* < 0.001 with Gaussian random field (GRF) theory for correction, and the cluster-level threshold was set to *p* < 0.05. The colour bar represents T values from the independent *t test*

Diminished ReHo was observed in the pooled MDD in the left postcentral gyrus and precentral gyrus (*p* < 0.001) relative to HCs using 2-tailed *t* tests (see Table 2 and Fig. 1).

#### Subgroup analysis

**Fig. 2:**
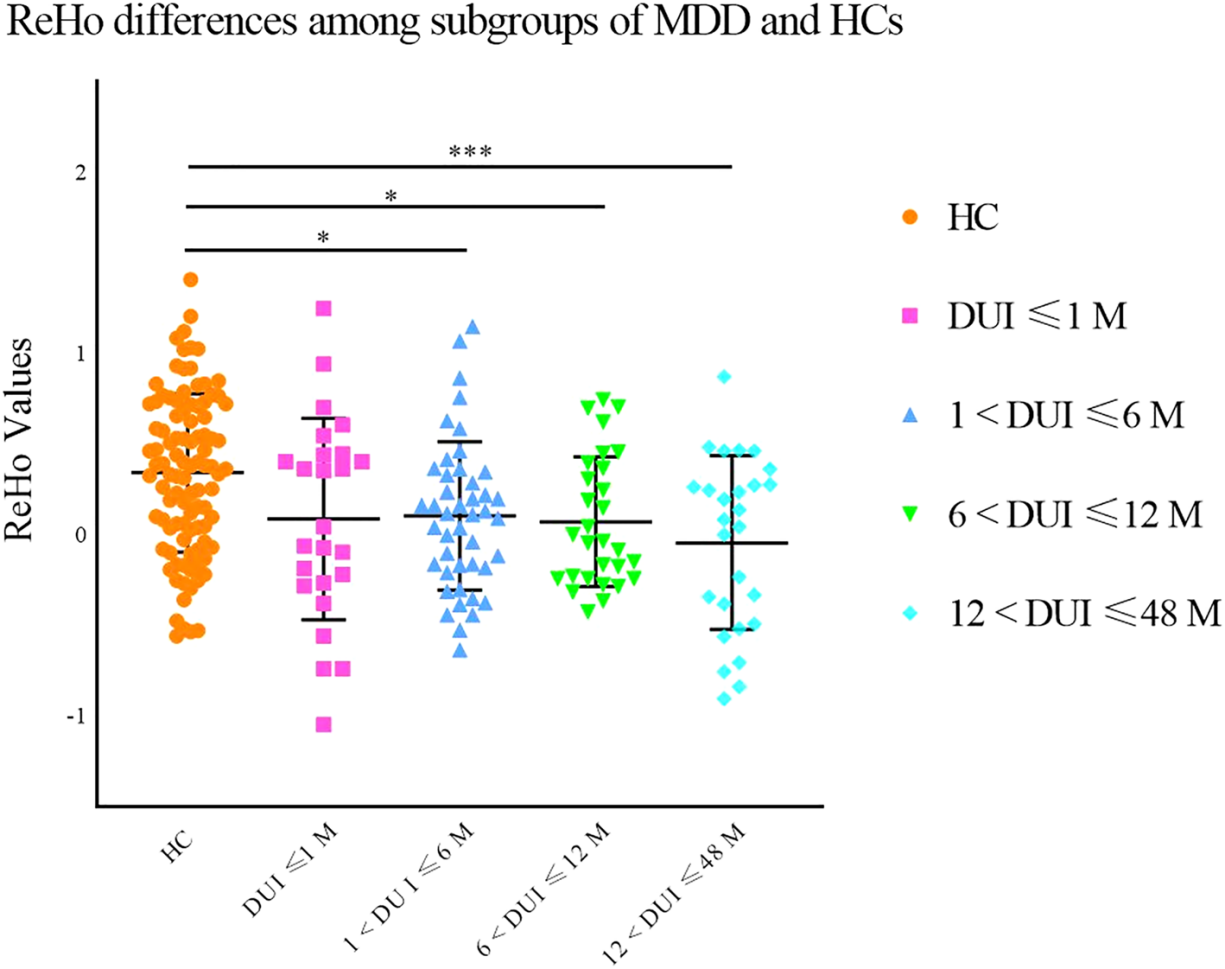
Significantly decreased ReHo in MDD patients with different DUIs in the left postcentral gyrus and the left precentral gyrus compared with HCs. Significant level was set to *p* < 0.05, with Bonferroni for post hoc correction. Note: 0.01 < *p* < 0.05 (*), 0.001 < *p* < 0.01 (**), *p* < 0.001 (***); DUI: duration of untreated illness

**Fig. 3:**
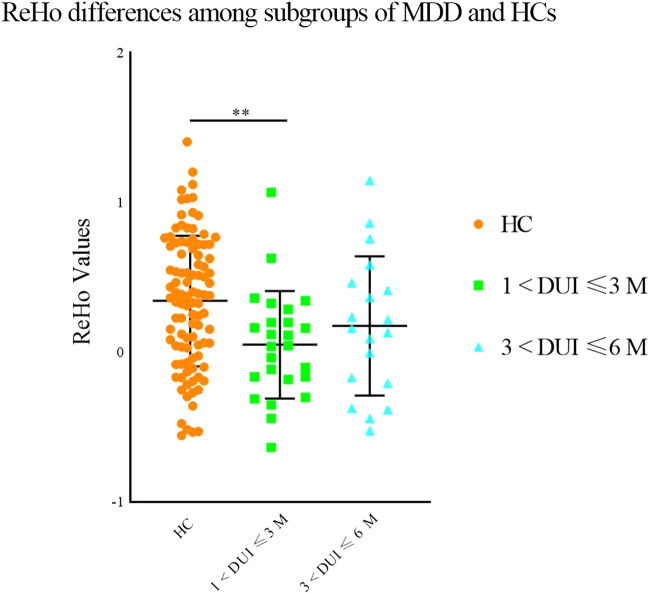
Significantly lower ReHo in MDD patients with different DUIs in the left postcentral gyrus and precentral gyrus compared with HCs. Significant level was set to *p* < 0.05, with Bonferroni for post hoc correction. Note: 0.01 < *p* < 0.05 (*), 0.001 < *p* < 0.01 (**); DUI: duration of untreated illness

The ReHo of HCs was not significantly different from that of MDD patients with a DUI less than 1 month. However, lower ReHo was observed in the left precentral and postcentral gyrus in MDD groups with a DUI longer than 1 month (1 < DUI ≤ 6 M: *p* = 0.032, 6 < DUI ≤ 12 M: *p* = 0.041, 12 < DUI ≤ 48 M: *p* < 0.001) compared to HCs. No significant differences were observed among the MDD subgroups (DUI ≤ 1 M, 1 < DUI ≤ 6 M, 6 < DUI ≤ 12 M, 12 < DUI ≤ 48 M, see Fig. 2). To identify the initial time point when ReHo abnormalities occurred, the subgroup of 1 < DUI ≤ 6 M was then allotted to 2 subgroups, i.e., 1 < DUI ≤ 3 M and 3 < DUI ≤ 6 M. Post hoc analysis revealed a significantly lower ReHo in the group of 1 < DUI ≤ 3 M (*p* = 0.008) compared with HCs. ReHo was higher in the group of 3 < DUI ≤ 6 M relative to the group of 1 < DUI ≤ 3, and no significant differences were detected between HCs and the MDD group of 3 < DUI ≤ 6 M or between the subgroups of 1 < DUI ≤ 3 M and 3 < DUI ≤ 6 M (see Fig. 3).

### Grey matter volume alterations

#### Pooled MDD versus HCs

**Fig. 4:**
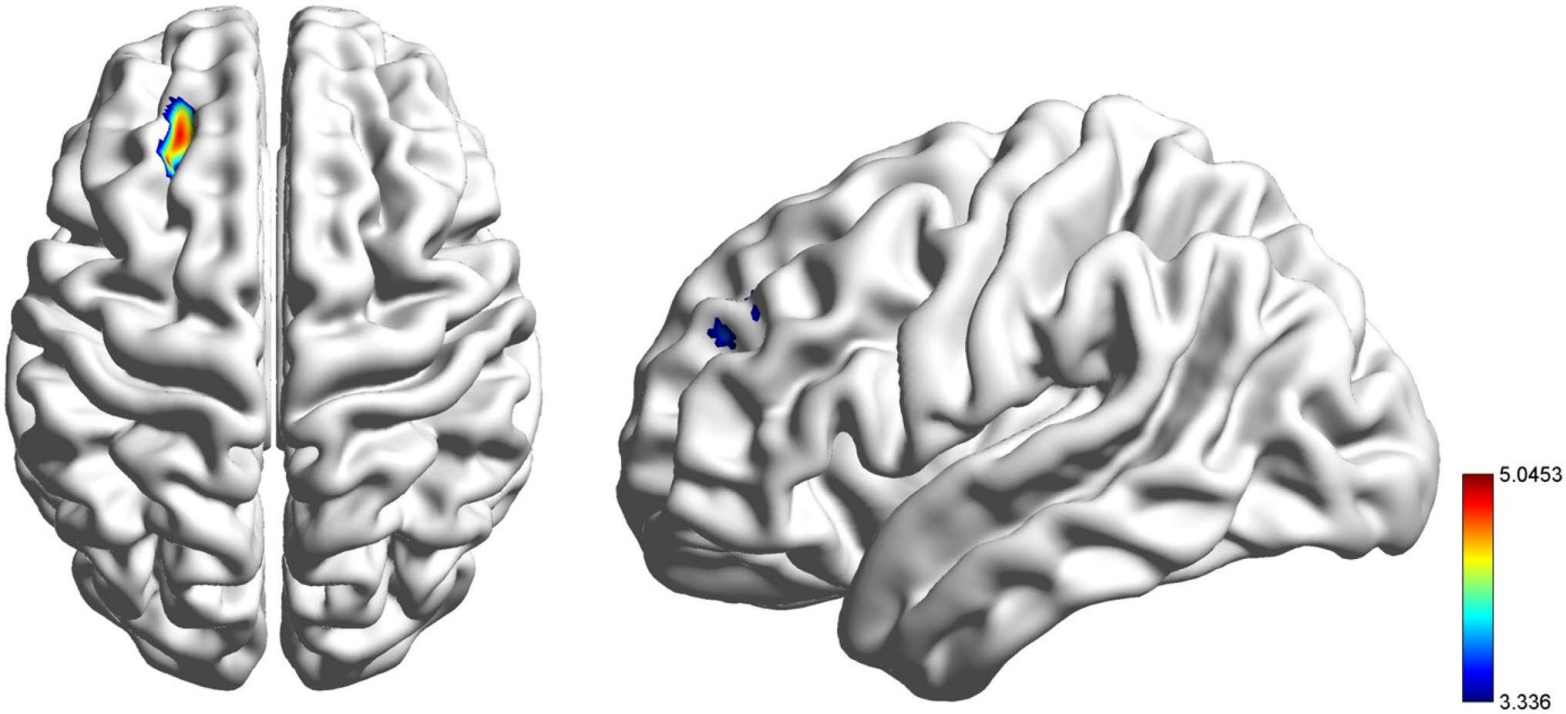
Significantly reduced grey matter volumes in the left middle frontal gyrus and superior frontal gyrus in the pooled MDD patients compared with HCs with 2-tailed *t test*. The significance of the voxel-level threshold was set to *p* < 0.001 with Gaussian random field (GRF) theory for correction, and the cluster-level threshold was set to *p* < 0.05. The colour bar represents T values from the independent *t test*

The pooled MDD was found to have lower GMV than HCs in the left middle frontal gyrus and superior frontal gyrus (*p* < 0.001) compared with HCs using general linear models, with TIV, age, age^2^, gender, and years of education as covariates (see Table 3 and Fig. 4).

**Table 3:** Clusters Showing Significant Differences in GMV Values between MDD and HC

Brain Regions (voxels)	Contrast	Hemisphere	Peak MNI Coordinates			F Values	*p*
			X	Y	Z		
Middle frontal gyrus (518)	HC > MDD	Left	− 25.5	31.5	42	3.336	< 0.001
Superior frontal gyrus (346)							

The pooled MDD patients and HCs were compared with general linear models

*GMV* Grey matter volume, *MDD* Major depressive disorder, *HCs* Healthy controls, *MNI* Montreal Neurological Institute

#### Subgroup analysis

**Fig. 5:**
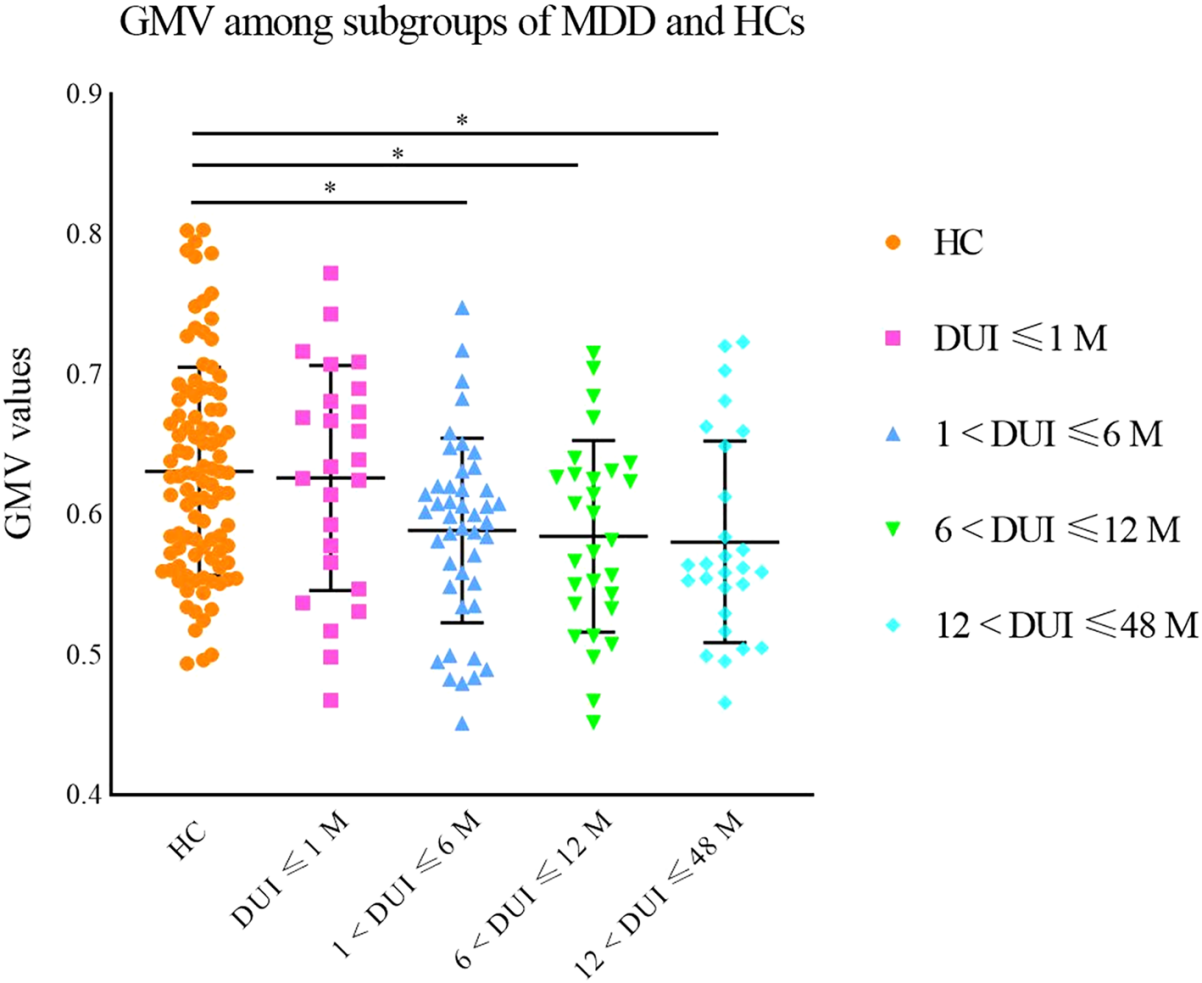
Significantly reduced grey matter volumes in the left middle frontal gyrus and superior frontal gyrus in MDD patients with different DUI compared with HCs. Significant level was set to *p* < 0.05, with Bonferroni for post hoc correction. Note: 0.01 < *p* < 0.05 (*); DUI: duration of untreated illness

**Fig. 6:**
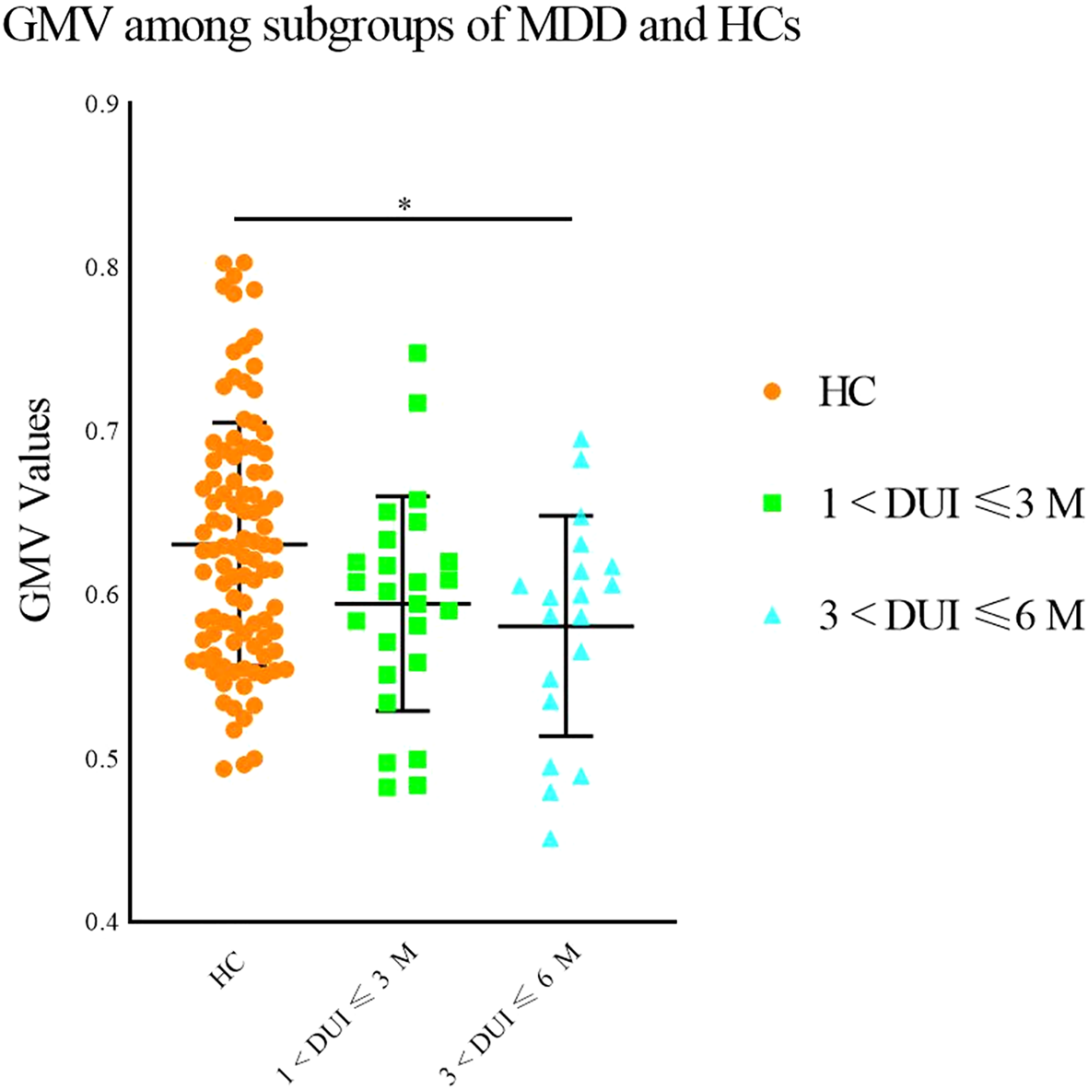
Significantly reduced grey matter volumes in the left middle frontal gyrus and superior frontal gyrus in MDD patients with different DUIs compared with HCs. Significant level was set to *p* < 0.05, with Bonferroni for post hoc correction. Note: 0.01 < *p* < 0.05 (*); DUI: duration of untreated illness

No significant abnormalities were discovered in GMV between HCs and MDD patients with DUIs shorter than 1 month. However, significantly lower GMV was found in the left middle frontal gyrus and superior frontal gyrus in MDD patients with DUIs longer than 1 month compared with HCs (1 < DUI ≤ 6 M: *p* = 0.015, 6 < DUI ≤ 12 M: *p* = 0.028, 12 < DUI ≤ 48 M: *p* = 0.016). No significant differences were obtained among the subgroups of MDD with different DUI (DUI ≤ 1 M, 1 < DUI ≤ 6 M, 6 < DUI ≤ 12 M, 12 < DUI ≤ 48 M), while a numerical trend was observed indicating lower GMV with longer DUI in MDD subgroups (see Fig. 5). To identify the earliest time point when GMV reduction occurred, the subgroup of 1 < DUI ≤ 6 M was then allotted to 2 subgroups as 1 < DUI ≤ 3 M and 3 < DUI ≤ 6 M. GMV was lower in the subgroup of 3 < DUI ≤ 6 M compared with HCs (*p* = 0.019), but no significant differences were discovered between HCs and the subgroup of 1 < DUI ≤ 3 M or between the subgroups of 1 < DUI ≤ 3 M and 3 < DUI ≤ 6 M (see Fig. 6).

### Relationships between GMV and clinical characteristics

**Fig. 7:**
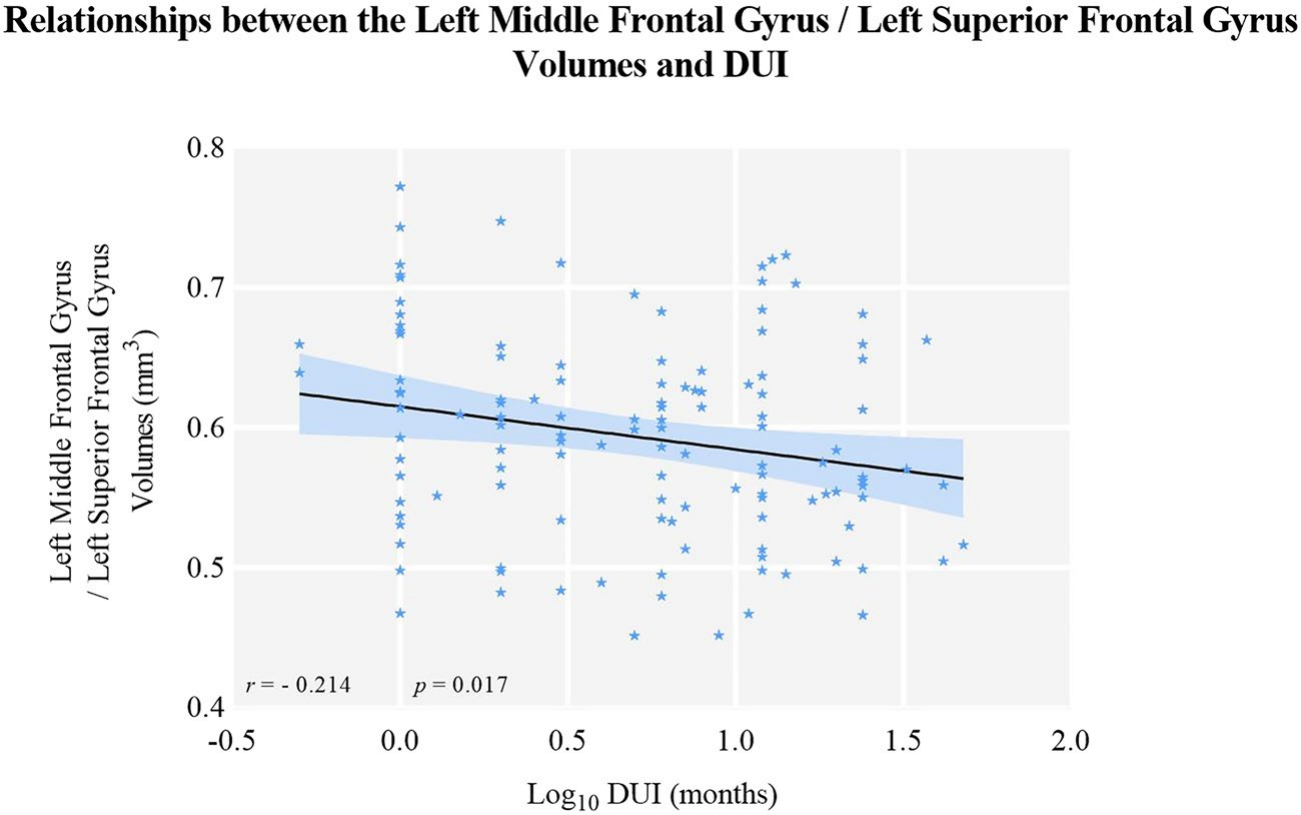
The left middle frontal gyrus/left superior frontal gyrus GMV were negatively related to the DUIs in MDD patients using Pearson correlation. Abbreviation: DUI: duration of untreated illness

**Fig. 8:**
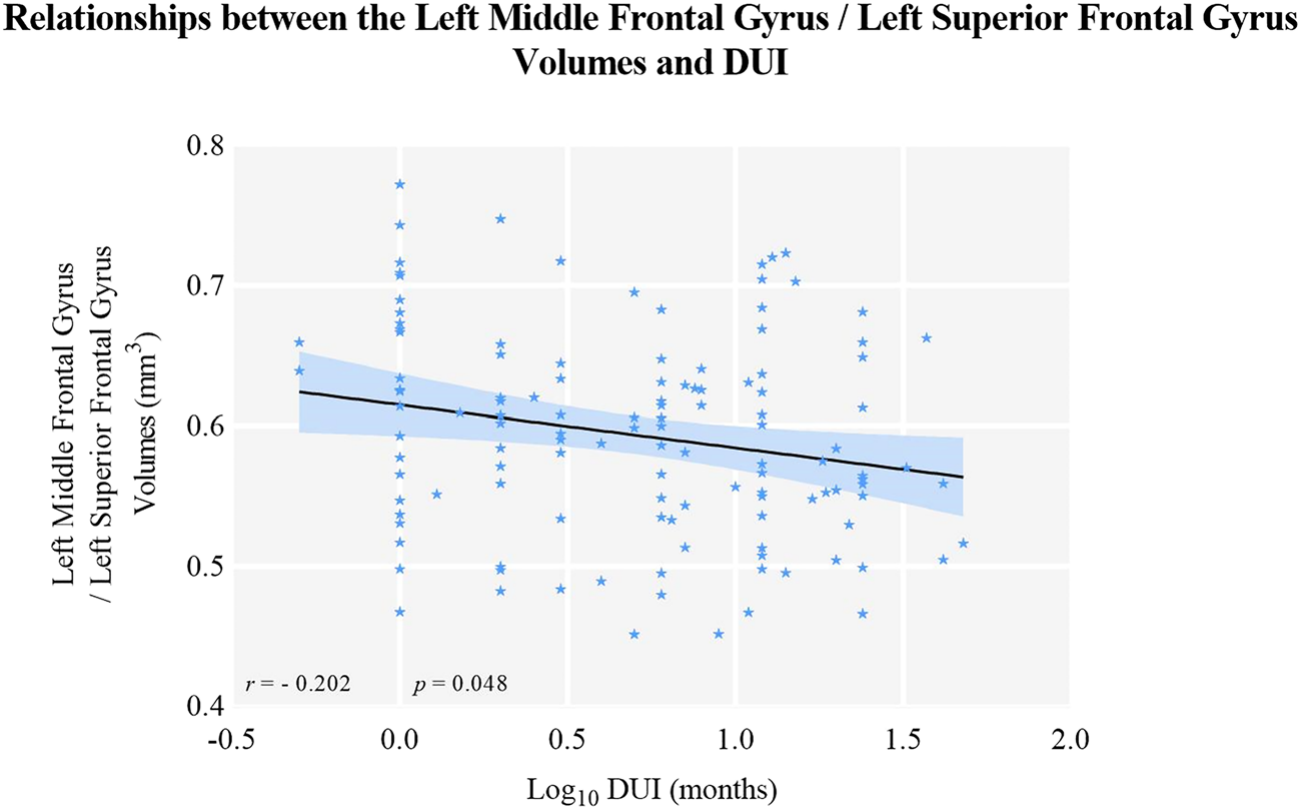
The left middle frontal gyrus/left superior frontal gyrus GMV were negatively related to the DUIs in MDD patients after controlling of the age of first onset. Abbreviation: DUI: duration of untreated illness

**Fig. 9:**
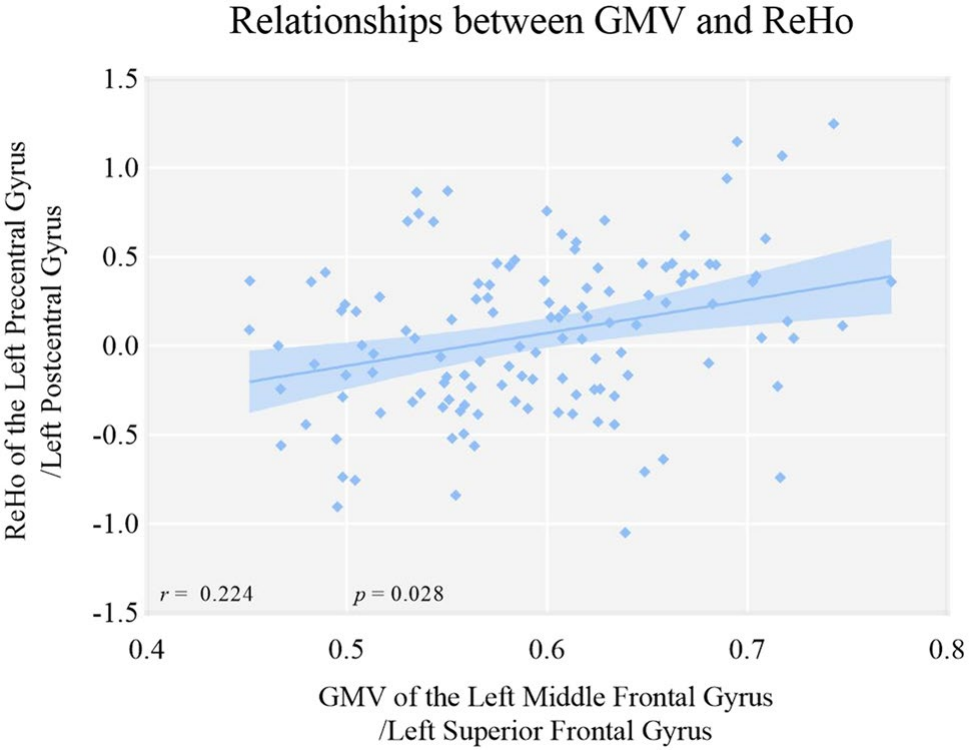
The left middle frontal gyrus/left superior frontal gyrus volumes were positively related to the ReHo in the left precentral/postcentral gyrus in MDD patients after controlling of the age of first onset

The left middle frontal gyrus and superior frontal gyrus volumes were negatively correlated with log_10_ DUI in MDD (*r* = -0.214, *p* = 0.017, q = 0.026, see Fig. 7 and Table 4). The GMV of the left middle frontal and superior frontal gyri was positively related to ReHo in the left precentral and postcentral gyri (*r* = 0.298, *p* = 0.001). The age of first onset was negatively correlated with both ReHo (*r* = -0.251, *p* = 0.005, q = 0.015) and GMV (*r* = -0.287, *p* = 0.001, q = 0.003), which was then taken as a covariate for partial correlation. GMV was positively related to ReHo (*r* = 0.224, *p* = 0.028) and negatively associated with log_10_DUI (*r* = -0.202, *p* = 0.048) after controlling for age of first onset (see Table 5, Fig. 8 and Fig. 9). However, the association between GMV and log_10_DUI failed to survive the FDR correction (q = 0.096).

**Table 4:** Correlations between Structural and Functional Alterations and Clinical Characteristics in MDD Patients

		Log_10_ DUI	Age of first onset	Number of previous episodes	WCST	HAMD-17	HAMA	YMRS
ReHo	*r*	− 0.056	− 0.251^*^	− 0.123	0.049	− 0.084	− 0.045	0.013
	*p*	0.535	0.005	0.173	0.634	0.353	0.62	0.887
	*q*	0.535	0.015	0.260	1.412	1.240	0.845	0.887
GMV	*r*	− 0.214^*^	− 0.287^**^	-0.08	0.112	0.011	− 0.031	0.11
	*p*	0.017	0.001	0.375	0.274	0.906	0.728	0.221
	q	0.026	0.003	0.375	0.971	0.548	0.906	0.884

**Table 5:** Partial Correlations between ReHo, GMV and Clinical Characteristics in MDD Patients

		Log_10_ DUI	Number of previous episodes	WCST	HAMD_17	HAMA	YMRS	ReHo
ReHo	*r*	− 0.067	− 0.102	− 0.16	− 0.054	− 0.01	− 0.029	1
	*p*	0.463	0.258	0.878	0.548	0.909	0.752	/
	q	0.463	0.516	1.171	2.192	0.909	1.504	/
GMV	*r*	− 0.202	− 0.085	0.022	0.116	0.072	0.027	0.224^*^
	*p*	0.048	0.409	0.83	0.258	0.484	0.797	0.028
	q	0.096	0.409	0.830	1.032	0.968	1.063	0.028

Partial correlation was conducted with age of first onset as covariate

WCST was computed with 97 participants; 0.01 < q < 0.05 (*)

*MDD* major depressive disorder, *ReHo* regional homogeneity, *GMV* grey matter volumes, *DUI* duration of untreated illness

No correlations were observed between ReHo and log_10_DUI. Neither ReHo nor GMV was related to clinical features with Pearson or partial correlations (number of previous episodes, WCST, HAMD_17, HAMA, and YMRS, see Table 4 and Table 5).

### Mediation effect between DUI, GMV, and ReHo

**Fig. 10:**
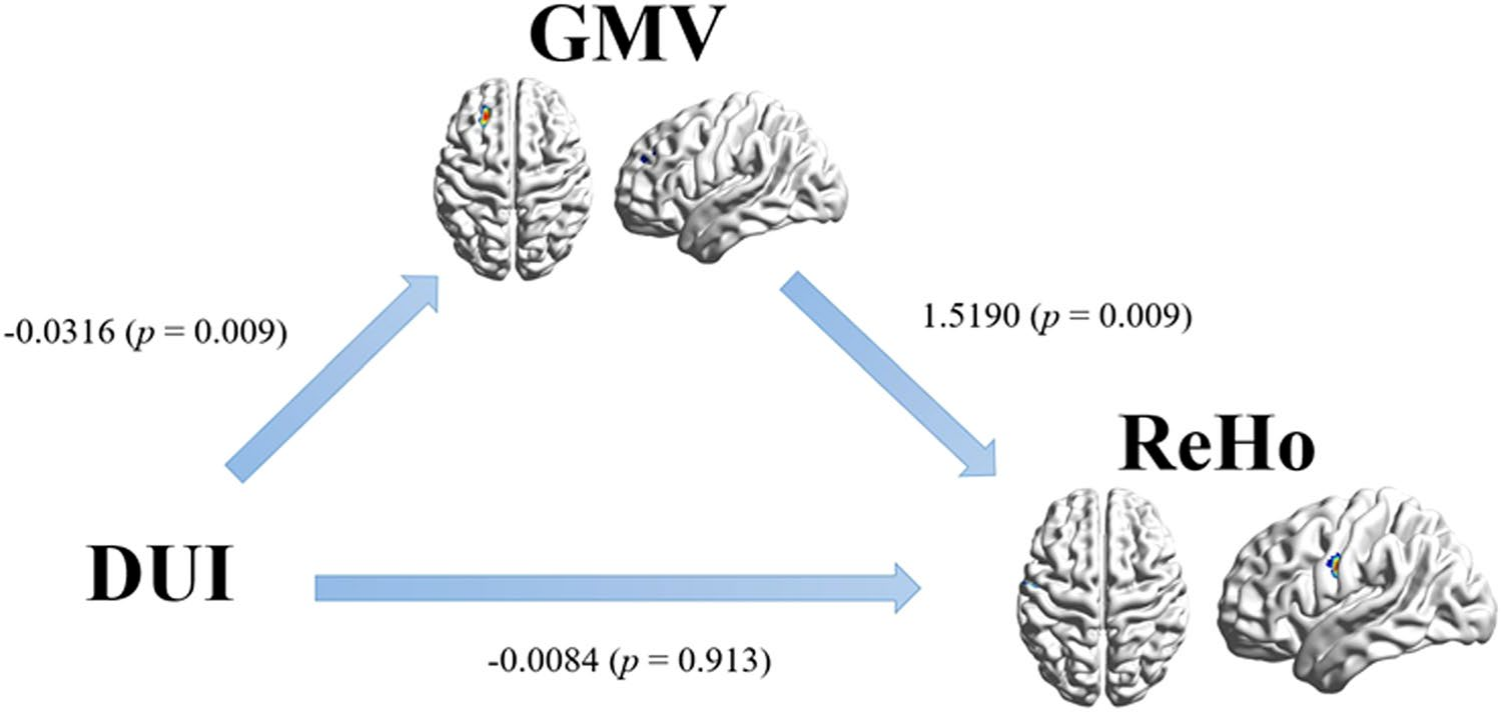
GMV in the left middle frontal gyrus/left superior frontal gyrus mediated the association between longer DUI and reduced ReHo in the left precentral/postcentral gyrus. Abbreviation: DUI: duration of untreated illness; GMV: grey matter volume; ReHo: regional homogeneity

After controlling for the age of first onset, 5000 bootstrapped samples revealed a significant indirect mediation effect (IE_GMV_ = -0.0480, 95% CI: LL = -0.1143, UL = -0.0056) from log_10_DUI to ReHo, with a mediator of GMV, indicating that MDD patients with longer DUI were more likely to have a reduced GMV and a consequent decline in ReHo. The ratio of the indirect effect to the total effect was 85.11%. The paths from log_10_DUI to GMV (β = -0.0316, SE = 0.012, *p* = 0.009) and from GMV to ReHo (β = 1.5190, SE = 0.5693, *p* = 0.009) were both significant. However, no significant direct effects were obtained from log_10_DUI to ReHo (β = -0.0084, SE = 0.077, *p* = 0.9130, see Fig. 10).

## Discussion

To our knowledge, this study is the first to explore the functional and structural abnormalities in different DUIs and to identify the initial time point when the neuroimaging alterations occurred in untreated MDD. We identified disassociated functional and anatomical alterations in treatment-naïve MDD patients at different time points in distinct brain regions at the early stage of the disease. Additionally, GMV mediated the relationship between longer DUI and diminished ReHo in MDD patients, implying a potential deleterious and neuro-progressive impact of DUI on the brain.

First, we discovered reduced ReHo in the left postcentral gyrus and precentral gyrus in treatment-naïve MDD patients, which initially occurred after being untreated for 1 month. In accordance with our findings, a decline in ReHo was also reported in the left precentral gyrus at the early stage, with a mean illness duration of 1.62 months [51]. In the subgroup analysis, lower ReHo was only observed in the subgroup of 1 < DUI ≤ 3 M, while no such phenomenon was identified in subgroup of 3 < DUI ≤ 6 M, but this value remained lower than that HCs, although not significantly, which may be due to compensatory effects. Kerestes et al. also reported an early-stage compensatory increase in functional connectivity in MDD patients with a median illness duration of 5 months [52]. Although we observed no further significant reduction in ReHo as the DUI became longer than 6 months, ReHo continuously remained lower in MDD patients than in HCs within this period. Therefore, we selected 6 months of being untreated as the time point for long-term functional deficiency of the brain. Previous studies also reported decreased ReHo in the frontal, temporal, parietal (bilateral precentral and postcentral gyrus included) and occipital cortices with a mean illness duration of approximately 6 months [53, 54]. We failed to identify the correlations between ReHo variations and DUI, but a previous study revealed a negative association between DUI and ReHo changes in MDD [55]. In this study, we observed that decreased ReHo was associated with longer DUI with GMV as mediator. Considering that the reduction in GMV occurred later than ReHo decreases, we speculate that the relationship between DUI and functional deficits was only mediated by structural alterations after being untreated for 6 months.

Furthermore, we identified reduced GMV in the left middle frontal gyrus and superior frontal gyrus in drug-naïve MDD patients, with 3 months of being untreated as the earliest time point of significant structural alterations. Our findings support the standpoint of MDD as a neuro-progressive illness [56] characterized by more severe pathological impairment with increasing illness durations [57, 58]. First-episode nonmedicated MDD was also previously associated with a significant GMV decrease in the left middle frontal gyrus and left superior frontal gyrus [59, 60], whereas the opposite observations were reported by Qiu et al. [61].

Although no significant differences in GMV were detected among MDD patients with different DUIs, we identified a numerical trend indicating lower GMV and longer DUIs in MDD patients. Lai discovered that lower GMV was associated with longer DUI [59], meanwhile, we failed to find that after FDR correction. In a recent study, whole-brain GMV was used for brain-predicted age difference (PAD), and PAD was demonstrated to be positively correlated with the DUI in MDD. This finding suggests more severe deficits in the GMV according to DUI, but only within the first 2 years of the episodes [62]. In addition, Han et. proposed the initial onset of the alteration as less than 3 months, unlike our proposed 3–6 months onset. This disparity in the initial time point may be due to the different brain regions we measured. Direct research on DUI and GMV is limited; however, studies on the relationships between neuroinflammation and DUI in MDD might suggest how DUI affects the brain. Neuroinflammation has been regarded as being associated with MDD [63, 64], the level of which was positively correlated with DUI [65] and negatively correlated with cortical thickness in the prefrontal regions [66, 67], indicating the deleterious inflammatory effect in MDD as DUI increased. Because antidepressants exert an anti-inflammatory effect [68], the modulation of regional activity in the fronto-limbic circuit, namely, the altered ReHo in the circuit disappeared [54] and the decreased metabolism in the prefrontal gyrus normalized [69] after treatment. Thus, we propose that early treatment can be beneficial to MDD patients.

## Limitation

First, our study was cross-sectional. Therefore, determining the progressive functional and anatomical alterations in different stages of DUI was difficult. A longitudinal study with reduplicated scans is essential to precisely detect the initial time point when the abnormalities occur. Second, the DUI was retrospectively attained from patients and their family members, and these data may consequently be subject to recall bias. Finally, the sample size of subgroups was small, and studies with a larger sample size are expected in the future.

## Conclusion

We were the first to identify a disassociated functional and structural alteration in treatment-naïve MDD patients at different time points in distinct brain regions at the early stage of the disease. Additionally, our analysis revealed that the relationship between longer DUI and reduced ReHo in MDD patients was mediated by GMV, disclosing the latent deleterious and neuro-progressive implications of DUI on both the structure and function of the brain and indicating the necessity of early treatment of MDD.

### Supplementary Information

Below is the link to the electronic supplementary material.

Supplementary file1 (DOCX 23 KB)
