# Supplementary material for: Functional and structural alterations in different durations of untreated illness in the frontal and parietal lobe in major depressive disorder
Source: Eur Arch Psychiatry Clin Neurosci. 2023 Aug 5;274(3):629–42. doi: 10.1007/s00406-023-01625-7 (PMC10995069; doi:10.1007/s00406-023-01625-7)
Supplement: Supplementary file 1 — Supplementary file1 (DOCX 23 KB) [file 406_2023_1625_MOESM1_ESM.docx]

**Supplementary**

**Table 1**

| **Demographic and Clinical Characteristics among MDD patients with Different DUI** | | | | | | |  |
| --- | --- | --- | --- | --- | --- | --- | --- |
|  | DUI ≤ 1 M  (n = 25) | 1 < DUI ≤ 6 M (n = 44) | 6 < DUI ≤ 12 M  (n = 29) | 12 < DUI ≤ 48 M (n = 27) | F / *χ2* | *p* |  |
| **Demographic characteristics** |  |  |  |  |  |  |  |
| Age at scan, years | 26.00 (9.31) | 27.66 (9.83) | 26.45 (8.75) | 27.41 (9.78) | 0.21 | 0.887 |  |
| Education, years | 13.36 (3.07) | 13.86 (2.81) | 14.21 (3.19) | 13.30 (3.05) | 0.59 | 0.625 |  |
| Male | 3 (12.00%) | 10 (22.73%) | 6 (20.69%) | 7 (25.93%) | 1.71 | 0.636 |  |
| Right handedness | 25 (100.00%) | 42 (95.45%) | 26 (89.66) | 25 (92.59) | 10.31 | 0.112 |  |
| **Clinical features** |  |  |  |  |  |  |  |
| Duration of un-treatment, months | 0.96 (0.14) | 3.73 (1.74) | 10.31 (2.16) | 24.32 (8.82) | 162.71 | < 0.001 |  |
| Number of previous episodes, times | 0.56 (0.51) | 0.64 (0.49) | 0.62 (0.49) | 0.89 (0.42) | 2.49 | 0.064 |  |
| Age of first onset, years | 26.04 (9.26) | 27.43 (9.70) | 25.38 (9.19) | 25.11 (10.17) | 0.43 | 0.732 |  |
| WCST | 32.43 (12.44) | 28.09 (10.12) | 29.00 (9.25) | 28.73 (9.38) | 0.82 | 0.484 |  |
| HAMD_17 | 18.88 (7.32) | 22.41 (8.98) | 22.24 (7.08) | 18.63 (6.07) | 2.23 | 0.088 |  |
| HAMA | 17.92 (8.57) | 20.16 (9.50) | 20.17 (7.46) | 17.70 (8.56) | 0.75 | 0.524 |  |
| YMRS | 1.00 (1.50) | 1.48 (2.03) | 1.34 (1.76) | 0.89 (1.34) | 0.834 | 0.478 |  |
| Note: Data are presented as either numbers (%) or means (standard deviations). ANOVA was conducted for continuous variables and chi-square test was conducted for categorical variables; Number of participants completed WSCT: DUI ≤ 1 M (n = 21); 1 < DUI ≤ 6 M (n = 34); 6 < DUI ≤ 12 M (n = 20); 12 < DUI ≤ 48 M (n = 22). Abbreviations: MDD: major depressive disorder; WCST: Wisconsin Card Sorting Test; HAMD-17: 17-item Hamilton Depression Rating Scale; HAMA: Hamilton Anxiety Rating Scale; YMRS: Young Mania Rating Scale; DUI: duration of untreated illness; M: month | | | | | | |  |
|  |  |  |  |  |  |  |  |
|  |  |  |  |  |  |  |  |
|  |  |  |  |  |  |  |  |

**Table 2**

| **Demographic and Clinical Characteristics among MDD patients with Different DUI** | | | | |  |
| --- | --- | --- | --- | --- | --- |
|  | 1 < DUI ≤ 3 M  (n = 25) | 3 < DUI ≤ 6 M  (n = 19) | *t* / *χ2* | *p* |  |
| **Demographic characteristics** |  |  |  |  |  |
| Age at scan, years | 26.96 (9.27) | 28.58 (10.72) | 0.29 | 0.594 |  |
| Education, years | 14.52 (2.80) | 13.00 (2.65) | 3.33 | 0.075 |  |
| Male | 3 (12.00%) | 7 (36.84%) | 3.79 | 0.051 |  |
| Right handedness | 24 (96.00%) | 18 (94.74%) | 0.4 | 0.842 |  |
| **Clinical features** |  |  |  |  |  |
| Duration of un-treatment, months | 2.33 (0.55) | 5.58 (0.69) | 301.28 | < 0.001 |  |
| Number of previous episodes, times | 0.56 (0.51) | 0.74 (0.45) | 1.44 | 0.237 |  |
| Age of first onset, years | 26.80 (9.06) | 28.26 (10.68) | 0.24 | 0.626 |  |
| WCST | 29.70 (10.31) | 24.73 (9.26) | 1.36 | 0.320 |  |
| HAMD_17 | 19.28 (7.45) | 26.53 (9.34) | 8.21 | 0.006 |  |
| HAMA | 17.60 (7.08) | 23.53 (11.29) | 4.55 | 0.039 |  |
| YMRS | 1.56 (1.83) | 1.37 (2.31) | 0.09 | 0.760 |  |
| Note: Data are presented as either numbers (%) or means (standard deviations). ANOVA was conducted for continuous variables and chi-square test was conducted for categorical variables; Number of participants completed WSCT: 1 < DUI ≤ 3 M (n = 23); 3 < DUI ≤ 6 M (n = 11); Abbreviations: MDD: major depressive disorder; WCST: Wisconsin Card Sorting Test; HAMD-17: 17-item Hamilton Depression Rating Scale; HAMA: Hamilton Anxiety Rating Scale; YMRS: Young Mania Rating Scale; DUI: duration of untreated illness; M: month. | | | | |  |
|  |  |  |  |  |  |
|  |  |  |  |  |  |
|  |  |  |  |  |  |
|  |  |  |  |  |  |
